# Effectiveness of Home-Based Cardiac Rehabilitation with Optimized Exercise Prescriptions Using a Mobile Healthcare App in Patients with Acute Myocardial Infarction: A Randomized Controlled Trial

**DOI:** 10.3390/life14091122

**Published:** 2024-09-05

**Authors:** Hyun-Seok Jo, Hyeong-Min Kim, Chae-Hyun Go, Hae-Young Yu, Hyeng-Kyu Park, Jae-Young Han

**Affiliations:** 1 Department of Physical & Rehabilitation Medicine, Chonnam National University Hospital, Gwangju 61469, Republic of Korea; jjm923@naver.com (H.-S.J.); freshsky986@naver.com (H.-M.K.); 2 Regional CardioCerebroVascular Center, Chonnam National University Hospital, Gwangju 61469, Republic of Korea; kon951@naver.com; 3 Biomedical Research Institute, Chonnam National University Hospital, Gwangju 61469, Republic of Korea; 154kg@naver.com; 4 Department of Physical & Rehabilitation Medicine, Regional CardioCerebroVascular Center, Chonnam National University Medical School & Hospital, Gwangju 61469, Republic of Korea; phk1118@naver.com; 5 Department of Physical & Rehabilitation Medicine, Regional CardioCerebroVascular Center, Center for Aging and Geriatrics, Chonnam National University Medical School & Hospital, Gwangju 61469, Republic of Korea

**Keywords:** cardiac rehabilitation, mobile applications, myocardial infarction, exercise prescription

## Abstract

Background: Despite the effectiveness of cardiac rehabilitation (CR), the actual participation rate in CR is low. While home-based CR offers a viable alternative, it faces challenges in participation due to factors such as a lack of self-motivation and fear of exercising without supervision. Utilizing a mobile healthcare application (app) during counseling may be an effective strategy for patients. Therefore, the aim of this study was to assess whether 6 weeks of home-based CR with exercise readjustment using a mobile app is an effective therapy for patients with acute myocardial infarction (AMI). Methods: Post-AMI patients eligible for home-based CR were randomized into the intervention group (CR-Mobile) and the control group, which followed the usual home-based CR protocol (CR-Usual). Both groups participated in a 6-week home-based CR program, with exercise readjustment and encouragement carried out every 2 weeks. The CR-Mobile group was supervised using data recorded in the mobile app, while the CR-Usual group was supervised via phone consultations. The primary outcome measured was maximal oxygen consumption (VO_2max_). Results: Within-group comparisons showed significant improvements in VO_2max_ (P_CR-Mobile_ = 0.011 vs. P_CR-Usual_ = 0.020) and METs (P_CR-Mobile_ = 0.011 vs. P_CR-Usual_ = 0.011) for both groups. Conclusions: These findings suggest that a 6-week home-based CR program with exercise readjustment using a mobile app can potentially enhance exercise capacity as effectively as verbal supervision.

## 1. Introduction

Cardiovascular disease (CVD) accounts for one-third of all deaths worldwide [1]. A similar trend is observed in Korea, where the mortality rate from CVD has increased by 42.8% over the past decade, making it the second leading cause of death in the country since 2014 [2]. Additionally, 62 per 100,000 people die from CVD, with 45% of these deaths resulting from ischemic heart disease [3]. Therefore, the prevention of CVD has long been emphasized. Cardiac rehabilitation (CR) is a critical component of secondary prevention. CR has been proven to decrease cardiovascular mortality and hospital readmissions [4]. Currently, patients participate in CR in 111 countries worldwide. However, despite its effectiveness, the actual participation rate in CR is low, ranging from 19% to 34% in the USA [5,6]. In South Korea, the participation rate is merely 1.5%, significantly lower than in many other countries [7]. Transportation limitations and travel distances are among the reasons for nonparticipation in CR [8]. Therefore, there have been attempts to overcome these barriers, with home-based CR suggested as an alternative. Previous studies have shown that home- and center-based CR appear to be equally effective in improving clinical and health-related quality of life outcomes in acute myocardial infarction (AMI) and revascularization patients [9]. However, home-based CR also faces challenges in participation due to factors such as a lack of self-motivation and fear of exercising without supervision [10]. Many clinical trials on home-based CR using smartphones have evaluated its efficacy. However, most have focused primarily on the functionalities of mobile apps, such as ease of use, exercise prescription reminders, encouragement, and educational content. One study revealed that self-motivation and family support help keep patients engaged in a home-based CR program [11]. Additionally, it is important for patients to have a sense of belonging [12]. To emphasize this point, counseling by healthcare professionals is crucial to enhance self-motivation. Therefore, in home-based CR, we expected that telemedicine interventions using smartphones would be more effective than conventional telephone-based approaches and could support center-based CR. Utilizing a mobile healthcare application (app) during counseling may be an effective strategy for patients, as it can help in setting the appropriate exercise time and intensity. Thus, additional benefits are expected with more personalized and optimized exercise prescriptions. Therefore, the aim of this study was to assess whether 6 weeks of home-based CR with exercise readjustment using a mobile app is an effective therapy method for patients with AMI.

## 2. Materials and Methods

### 2.1. Study Design

**Figure 1:**
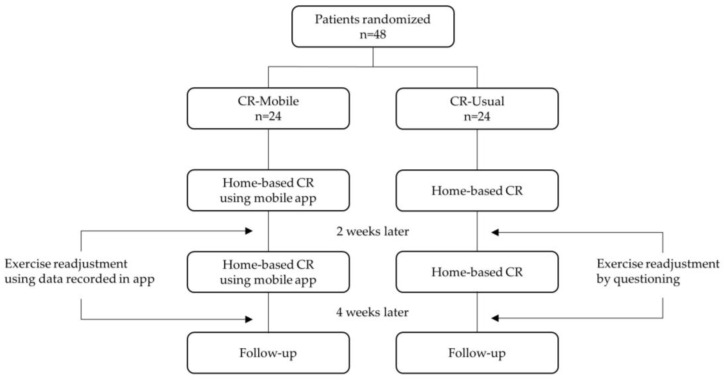
Study flowchart.

This single-blind, randomized controlled study was performed at a single center. The study evaluated the efficacy of home-based CR with exercise readjustment using a mobile app in patients with AMI. Forty-eight participants were recruited from the outpatient clinic of Physical Medicine & Rehabilitation at Chonnam National University Hospital. Post-AMI patients eligible for home-based CR were randomized into the intervention group (CR-Mobile, n = 24) and the control group, which received the usual home-based cardiac rehabilitation (CR-Usual, n = 24). Both groups participated in a 6-week home-based CR program, with exercise readjustment carried out every 2 weeks. The study flowchart is presented in Figure 1.

**Figure 2:**
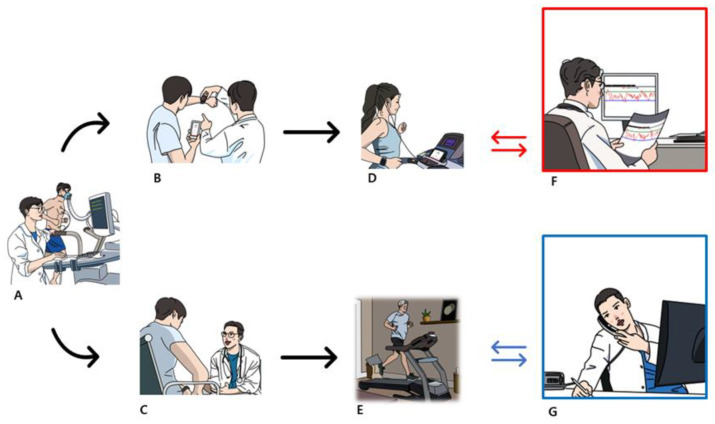
Flow of the study: (**A**) patients performing the exercise tolerance test, (**B**,**C**) exercise prescription either through a mobile app or verbally, (**D**,**E**) home-based cardiac rehabilitation with or without a mobile app, (**F**) exercise readjustment through the data recorded in the mobile app every 2 weeks, (**G**) exercise readjustment by questioning the patients over the phone every 2 weeks.

A symptom-limited exercise tolerance test (ETT) was performed using the modified Bruce protocol at enrollment. A treadmill (Med-Track ST 55; Quinton Instruments, Seattle, WA, USA) was used during the ETT. After that, both groups were educated about cardiac rehabilitation, including the exercise program, lifestyle modification, and risk factor management. Exercise intensity was prescribed based on the target heart rate, calculated using the Karvonen formula, and personalized according to risk stratification [13]. Both groups were prescribed exercise three times per week. The CR-Mobile group received an exercise prescription utilizing a mobile app during their first visit, while the CR-Usual group received a prescription without any explanation related to the mobile app. Every 2 weeks, the CR-Mobile group was supervised through the data recorded in the mobile app (Harufit Cardio, L&H Labs Inc., Gwangju, Republic of Korea), while the CR-Usual group was supervised by questioning the patients over the phone. The supervision involved questioning the Rating of Perceived Exertion (RPE) Borg scale, as it was not possible to accurately monitor heart rate, and checking the frequency, time, and type of exercise (Figure 2).

### 2.2. Mobile Application

**Figure 3:**
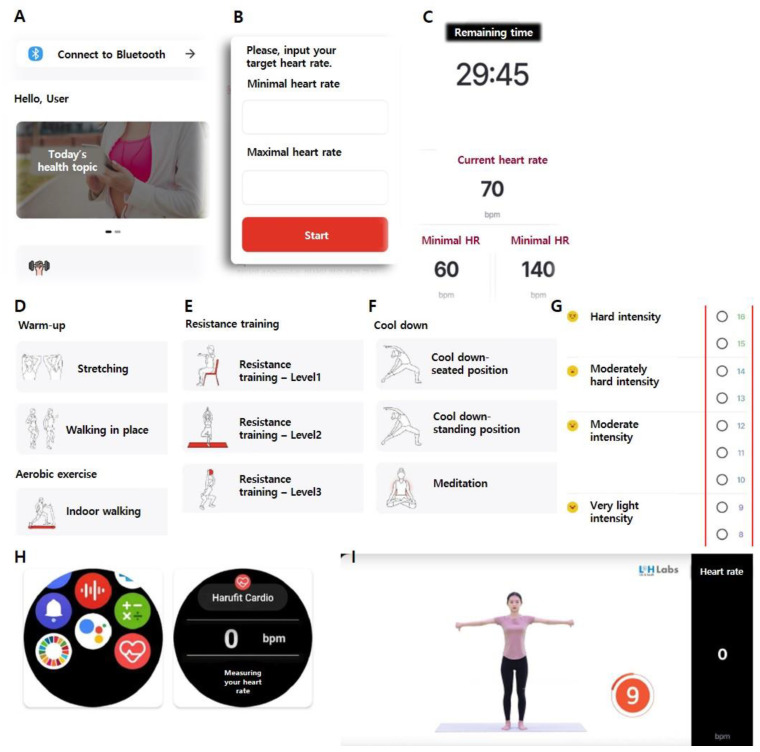
Featured smartphone application. (**A**) Main menu displays users and exercise data. (**B**) Display of target heart rate setting. (**C**) Timer and real-time heart rate of participants during exercise, with target heart rate shown below. (**D**–**F**) Exercise menu, type of exercise (warm-up, aerobic, resistance, stretching exercise, and cool down) are displayed, and the user can select the program. (**G**) Rating of perceived exertion (RPE) Borg scale is displayed. Participants can choose after exercise. (**H**) Wearable smartwatch screen mobile app records real-time heart rate. (**I**) Instruction videos.

**Figure 4:**
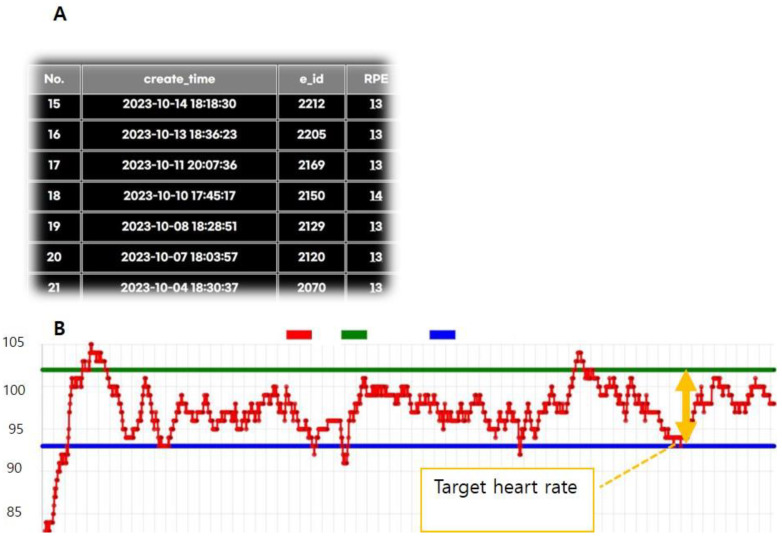
Web platform for healthcare professionals. (**A**) Daily records of participants’ exercise and rating of perceived exertion (RPE) Borg scale are displayed. When healthcare professionals click the date, (**B**) they can see the heart rate of participants during exercise, as the red line. The green and blue lines are the target heart rates (maximal and minimal) prescribed by physicians.

The mobile application (Harufit) was developed by the corresponding author and L&H Labs Inc. (Gwangju, Republic of Korea) on an Android platform. They continuously discussed the application through numerous meetings, with the corresponding author providing information about the entire exercise program and guidelines. Considering the age of the participants, the application focused on user-friendly interfaces. The application included exercise modes for warm-up, aerobic, stretching, and resistance exercises, and it provided exercise records and a diary. The mobile app can pair with a wearable smartwatch via Bluetooth (Figure 3). If the heart rate of participants was not within the target range, the smartwatch provided a vibration alert. Additionally, a web platform was developed to monitor the data, allowing healthcare professionals to check patients’ exercise data in real-time (Figure 4).

### 2.3. Intensity and Volume of Exercise Protocol

Based on the American Association of Cardiovascular and Pulmonary Rehabilitation [13] and the ninth edition of ACSM’s guidelines [14], exercise intensity and volume were determined. Exercise started with 5–10 min of warm-up, followed by 30 min of aerobic exercise (fast walking, ground running, cycling, or other exercises to meet the target heart rate). This was followed by 10 min of resistance or stretching exercises, and the session ended with 5 min of cool down. Participants chose the RPE Borg scale (6 to 20), and healthcare professionals could see the data on the web platform. If the RPE Borg scale was not between 11 and 13, healthcare professionals adjusted the exercise intensity, time, or frequency [15]. Additionally, if the heart rate of participants was not within the target heart rate range during exercise, healthcare professionals re-educated them about exercise intensity.

### 2.4. Study Participants and Randomization

Post-AMI patients eligible for home-based CR were screened by physicians. The inclusion criteria were as follows: (1) aged 19–75 years, (2) post-AMI patients eligible for home-based CR, (3) low to moderate risk for CR by risk stratification [13], (4) independent walking, and (5) having a smartphone. Patients were excluded if they (1) had contraindications for CR, (2) were unsuitable to participate in the study due to a medical condition, (3) were pregnant, (4) lacked the ability to read and speak Korean, (5) did not have a smartphone compatible with the app, or (6) refused consent. At enrollment, participants were allocated to the CR-Mobile or CR-Usual group with a 1:1 ratio. Group allocation was determined using random allocation software. Research coordinators were informed of the allocation only after it was determined. The investigators were blinded to the group allocation until the end of the study.

### 2.5. Sample Size Calculation

G*Power 3.1 (Heinrich Heine University Düsseldorf, Düsseldorf, Germany) was used to determine the number of participants needed [16]. Based on an expected difference in the VO_2max_ increase of 3.5 mL/kg/min in the intervention group [17,18,19,20], the effect size of 0.875 was derived from the mean power and standard deviation from a previous study [21]. To achieve an α of 0.05 and a power of 80%, at least 22 patients were required for each group. Considering a 10% dropout rate, a total of 48 patients, 24 in the CR-Mobile group and 24 in the CR-Usual group, were needed.

### 2.6. Measurements of Variables

#### 2.6.1. Patient Characteristics

Participants’ baseline characteristics were collected, including sex, age, height, Body Mass Index, underlying diseases, left ventricular ejection fraction, and history of coronary artery reperfusion therapy.

#### 2.6.2. Outcome Variables

The study outcomes were estimated from exercise tolerance tests at baseline and 6 weeks from the baseline assessment. Exercise capacity was determined by measuring VO_2max_ (ml/kg/min) and metabolic equivalents (METs) using an integrated metabolic measurement system (TrueOne 2400 Metabolic System, Ver. 4.0.1.458 SP1; Parvo-Medics, Sandy, UT, USA) during the exercise tolerance test. Secondary outcomes including resting heart rate (HRrest), maximal heart rate (HRmax), resting systolic blood pressure (SBP), and resting diastolic blood pressure (DBP) were measured using an automatic blood pressure and pulse monitor. RPE was recorded using the Borg scale of 6–20 points.

Quality of life was evaluated using the EuroQol 5-Dimension 5-Level (EQ-5D-5L) [22]. The ability to perform specific activities was evaluated using the Korean Activity Scale/Index (KASI) [23]. Mental health was assessed using the Patient Health Questionnaire-9 (PHQ-9) [24].

### 2.7. Statistical Analysis

A data analysis was performed using SPSS Statistics for Windows, version 21 (IBM Corp., Armonk, NY, USA). Outcomes at the end of the 6-week home-based CR were compared with the baseline. Continuous variables were presented as mean ± standard deviation and analyzed using independent *t*-tests. Categorical variables were presented as numbers (percentages) and analyzed using the chi-square test. Group comparisons were made using paired *t*-tests. A repeated measures analysis of variance was performed to evaluate the effects within-group and between-groups over time. A *p*-value less than 0.05 was considered statistically significant.

### 2.8. Ethics Statements

Ethical approval for the study protocol was granted by the Institutional Review Board of Chonnam National University Hospital (IRB No. CNUH-EXP-2023-222). Informed consent was obtained from all participants. This study complied with the guidelines of the Declaration of Helsinki.

## 3. Results

### 3.1. Demographic Characteristics

A total of 48 participants were enrolled, but 7 were lost to follow-up due to the distance from their residence. Participants’ demographic data are shown in Table 1. Overall, the participants were middle-aged (mean age, 57.0 ± 9.9 years), and the majority were male (93.8%). The BMI was slightly above normal (25.3 ± 3.1). Regarding comorbidities, 39% had hypertension (HTN), 26% had diabetes mellitus (DM), and 39% had dyslipidemia (DL). The average left ventricular ejection fraction (LVEF) of the participants was 53.6 ± 9.7%, and most participants had a history of percutaneous coronary intervention (PCI) (97.9%). There were no significant differences in patient characteristics and the index event.

### 3.2. Outcome Parameters

Table 2 shows the primary and secondary outcome parameters at baseline and at the 6-week follow-up. Diastolic and systolic blood pressures showed no significant differences between the two groups at baseline. After the intervention, there were no significant differences in both the between-group and within-group comparisons. In the within-group comparison, both groups showed significant improvement in VO_2max_ (P_CR-Mobile_ = 0.011 vs. P_CR-Usual_ = 0.020) and METs (P_CR-Mobile_ = 0.011 vs. P_CR-Usual_ = 0.011). However, in the between-group comparison, no significant difference was found between the groups (P_time x group_ = 0.947). There were no significant differences between the two groups in their resting and maximum heart rate at baseline, and no differences were observed between the groups after the intervention. Additionally, there was no significant difference in the change in physical activity level, psychological well-being, and quality of life status between the two groups.

## 4. Discussion

This study demonstrated that using a mobile app for exercise readjustment in home-based CR has a favorable effect on improving the exercise capacity of patients with AMI. CR is a class 1A recommended part of cardiac care for patients with cardiovascular disease [25]. The benefits of CR for patients with AMI have been unquestionably proven [26]. A recent study also demonstrated a dose–response relationship between CR programs and mortality [27]. However, between 24 and 50% of patients who enroll in CR drop out, which is suboptimal [28]. Efforts to improve adherence to CR have continued, and a previous study suggested that even minimal counseling by medical professionals each week can impact adherence [29]. Therefore, we focused on minimal counseling. Most smartphone-based CR studies have focused on aspects related to ease of use for home-based exercise programs and the role of reminders in enhancing compliance [30]. However, even in home-based CR, minimal counseling adjusted to the patient’s condition should be considered more effective for increasing participation rates than mere ease of use. Therefore, we conducted exercise readjustment every two weeks for both the CR-Mobile group and the CR-Usual group. The CR-Mobile group received exercise counseling based on data recorded in the mobile app, while the CR-Usual group received exercise counseling based on questionnaires, and we compared the differences in outcomes accordingly.

We found that both groups showed significant improvement in VO_2max_ and METs. Traditionally, home-based CR has been proven to be as effective as center-based CR [9]. One study showed that important patient expectations about home-based CR included communication with specialists and making periodic visits to maintain motivation and adhere to the home-based CR programs [31]. We conducted communication in one group using a mobile app and in the other group through verbal questioning, and the difference in exercise capacity between the groups was not significant. Therefore, for effective home-based CR, the involvement of cardiac rehabilitation specialists would be necessary. Additionally, considering the improvement in VO_2max_ and METs observed in both groups, it is expected that real-time counseling and exercise data monitoring contributed to an increase in self-motivation among the patients. The Δchange in VO_2max_ in the CR-Mobile group was 3.2 mL/kg/min and 3.3 mL/kg/min in the CR-Usual group. Considering that an increase of 1 mL/kg/min VO_2max_ is associated with approximately a 10% reduction in cardiovascular mortality [32], exercise readjustment using a mobile app can be effective for patients. Many other studies have compared home-based CR using mobile apps versus center-based CR or conducted prospective single-arm studies using just the mobile app group. However, we conducted a study in which the control group and the intervention group had the same conditions, except for the exercise readjustment method. Therefore, we can more strongly suggest that readjustment performed through comprehensive telerehabilitation using data recorded in a mobile app is effective for improving exercise capacity. Additionally, the time for exercise readjustment can be reduced, and counseling can be conducted with more objective data.

A concern during this study was that the mean age of the CR-Mobile group was 58 years. Regarding digital literacy in older adults, South Korea has achieved a high rate of smartphone penetration (97.1%), even among individuals 60 years old and above (90%) [33]. However, the digital literacy rate among adults aged 55 and older is only 64.3%, with those aged 70 and older notably lower at 35.7% in South Korea [34]. Therefore, when considering the use of a mobile app for home-based CR in terms of digital literacy, there was a concern that it might have a negative impact; however, the results between the two groups were actually similar. Therefore, a mobile app can be a good treatment option for older patients.

There were no significant differences in physical activity level, psychological well-being, and quality of life status in the between-group and within-group comparisons. Generally, home-based CR can improve quality of life and psychological state and can help control risk factors for coronary heart disease [35]. In our study, the physical activity level, psychological well-being, and quality of life status scores at baseline were already nearly normal. One study suggested that the self-motivation of patients helps to keep them engaged in home-based CR [11]. Therefore, patients who were already physically and mentally healthy may have participated in our study, leading to no significant differences in the results within or between groups. Further studies with a larger and more diverse spectrum of patients are needed.

There were no significant differences in blood pressure and heart rate after the 6-week intervention. One study showed that a 2-month exercise rehabilitation program in post-myocardial infarction patients is useful for improving both blood pressure and exercise capacity [36]. In our study, most participants were taking medication. Also, measuring blood pressure and heart rate at those two time points may not accurately reflect the changes. We were unable to implement real-time vital sign monitoring in the mobile app due to privacy regulations. In the future, it will be necessary to monitor continuous changes in vital signs to better understand the effects of exercise.

Home-based CR using a mobile app enables real-time counseling and exercise data monitoring between medical professionals and patients, facilitating individualized exercise readjustments. Ultimately, home-based cardiac rehabilitation with optimized exercise readjustment using a mobile app can improve patients’ functional capacity and may increase adherence. Moreover, considering the home environment, this approach can be particularly effective during situations like the COVID-19 pandemic when facility access is limited.

There were several limitations to our study. First, the sample size was small, and all of the patients were recruited from one center. As a result, it is difficult to generalize the statistical results. Thus, a multicenter study is needed to verify our results of home-based CR with optimized exercise readjustment using a mobile app. By including multiple countries, our results will be more applicable to other regions. Second, our study did not consider the maintenance of the effect of home-based CR after the 6-week intervention. When initially designing the study, we planned to conduct a follow-up exercise tolerance test after 12 weeks. However, considering dropout rates, we designed the study to last 6 weeks. Future studies with a greater number of patients and long-term follow-up are needed to determine if the effects last beyond 6 weeks. Third, we did not check the lipid profile, for example, the lipoprotein and triglyceride levels. Because lipoprotein is associated with cardiovascular mortality [37], it will be necessary to include laboratory assessments in future studies. Additionally, we did not include variables related to muscle mass index or grip strength. Although our exercise protocol included resistance training, further studies will be required to determine the effects of resistance training.

## 5. Conclusions

These results suggest that a 6-week home-based CR with exercise readjustment using a mobile app for patients with AMI is demonstrated to improve exercise capacity as effectively as verbal supervision. Although significant differences were not observed between the groups, further large-scale multicenter studies, including a group that is not supervised for exercise readjustment, are needed.

## Figures and Tables

**Table 1:** Demographic characteristics of participants.

	Total (n = 48)	CR-Mobile (n = 24)	CR-Usual (n = 24)	*p*-Value
Age (years)	57.0 ± 9.9	58.0 ± 12.4	56.0 ± 7.0	0.501
Sex, no				0.551
Male (%)	45 (93.8)	22 (91.7)	23 (95.8)	
Female (%)	3 (6.3)	2 (8.3)	1 (4.2)	
BMI (kg/m^2^)	25.3 ± 3.1	25.1 ± 2.6	25.6 ± 3.6	0.581
LVEF (%)	53.6 ± 9.7	54.7 ± 11.3	52.5 ± 8.0	0.441
Comorbidities				
HTN (%)	18 (39.1)	10 (43.5)	8 (34.8)	0.546
DM (%)	12 (26.1)	5 (21.7)	7 (30.4)	0.502
DL (%)	18 (39.1)	7 (30.4)	11 (47.8)	0.227
Index intervention				0.312
PCI	47 (97.9)	24 (100)	23 (95.8)	
PCI + CABG	1 (2.1)	0 (0)	1 (4.2)	

Values are presented as mean ± standard deviation. Test statistics are presented as χ2 or t value. Abbreviations: CR-Mobile, home-based cardiac rehabilitation supervised by a mobile healthcare application; CR-Usual, home-based cardiac rehabilitation verbally supervised; LVEF, left ventricular ejection fraction; HTN, hypertension; DM, diabetes mellitus; DL, dyslipidemia; PCI, percutaneous coronary intervention; CABG, coronary artery bypass surgery.

**Table 2:** Primary and secondary outcome parameters at baseline and 6-week follow-up.

	CR-Mobile Group (n = 20)			CR-Usual Group (n = 21)			Mobile vs. Usual Comparison	
	Baseline	Post (6 Weeks)	*p*-Value	Baseline	Post (6 Weeks)	*p*-Value	Time	Time × Group
VO_2max_ (mL/kg/min)	27.34 ± 3.86	30.52 ± 7.24	0.011	25.75 ± 7.18	29.05 ± 9.96	0.020	0.001	0.947
METs	7.8 ± 1.1	8.7 ± 2.1	0.011	7.4 ± 2.1	8.3 ± 2.8	0.011	0.001	0.947
HR_rest_ (beats/min)	66.7 ± 6.8	68.4 ± 4.7	0.255	69.9 ± 5.2	70.5 ± 6.7	0.648	0.251	0.604
HR_max_ (beats/min)	143.7 ± 14.4	147.2 ± 19.6	0.403	145.2 ± 16.8	146.0 ± 23.2	0.825	0.434	0.624
SBP_rest_ (mmHg)	114.8 ± 13.3	116.8 ± 18.7	0.584	122.2 ± 18.6	128.2 ± 16.0	0.078	0.103	0.400
DBP_rest_ (mmHg)	73.7 ± 9.8	74.1 ± 10.9	0.857	72.7 ± 11.1	77.0 ± 14.1	0.258	0.198	0.285
SBP_max_ (mmHg)	161.9 ± 30.3	171.5 ± 28.1	0.191	177.9 ± 28.5	164.2 ± 35.9	0.127	0.718	0.044
DBP_max_ (mmHg)	74.0 ± 10.4	79.7 ± 18.0	0.118	82.6 ± 17.6	82.9 ± 16.3	0.513	0.2100	0.247
KASI	54.9 ± 8.3	57.9 ± 6.4	0.195	54.6 ± 7.0	59.4 ± 4.3	0.004	0.005	0.502
EQ-5D	5.2 ± 0.6	5.5 ± 0.6	0.332	5.4 ± 0.7	5.6 ± 1.2	0.565	0.291	0.814
PHQ-9	0.3 ± 1.3	0.4 ± 1.0	0.713	0.1 ± 0.3	0.2 ± 0.5	0.655	0.669	0.863

Values are presented as mean ± standard deviation. The effects of cardiac rehabilitation on the endpoints were analyzed using paired *t*-tests. Repeated measures analysis of variance was conducted to examine within-group and between-group over-time differences. A *p*-value < 0.05 was considered to be of statistical significance. Abbreviations: CR-Mobile, home-based cardiac rehabilitation supervised by a mobile healthcare application; CR-Usual, home-based cardiac rehabilitation verbally supervised; VO_2max_, maximal oxygen consumption; METs, metabolic equivalents; HRrest, resting heart rate; HRmax, maximal heart rate; SBPrest, resting systolic blood pressure; DBPrest, resting diastolic blood pressure; SBPmax, maximal systolic blood pressure; DBPmax, maximal diastolic blood pressure; KASI, Korean Activity Scale/Index; EQ-5D, EuroQoL-5 dimensions; PHQ-9, Patient Health Questionnaire-9.

## Data Availability

All the data analyzed during the study are included in this manuscript.
